# Redesign, Field-Testing, and Validation of the Physical Activity Campus Environmental Supports (PACES) Audit

**DOI:** 10.1155/2019/5819752

**Published:** 2019-05-19

**Authors:** Tanya M. Horacek, Elif Dede Yildirim, Dean Seidman, Carol Byrd-Bredbenner, Sarah Colby, Adrienne A. White, Karla P. Shelnutt, Melissa D. Olfert, Anne E. Mathews, Kristin Riggsbee, Lisa Franzen-Castle, Jesse Stabile Morrell, Kendra Kattelmann

**Affiliations:** ^1^Department of Public Health Food Studies and Nutrition, Syracuse University, Syracuse, NY 13244, USA; ^2^Human Development and Family Studies, Auburn University, Auburn, AL 36849, USA; ^3^Abramson Center for Jewish Life, North Wales, PA 19454, USA; ^4^Department of Nutritional Sciences, Rutgers University, New Brunswick, NJ 08901, USA; ^5^Department of Nutrition, University of Tennessee, Knoxville, TN 37996, USA; ^6^School of Food and Agriculture, University of Maine, Orono, ME 04469-5735, USA; ^7^Department of Family, Youth and Community Sciences, University of Florida, Gainesville, FL 32611, USA; ^8^Division of Animal & Nutritional Sciences, School of Agriculture, West Virginia University, Morgantown, WV 26506, USA; ^9^Food Science and Human Nutrition Department, University of Florida, Gainesville, FL 32611, USA; ^10^Department of Nutrition and Health Sciences, University of Nebraska-Lincoln, Lincoln, NE 68566, USA; ^11^Department of Agriculture, Nutrition and Food Systems, University of New Hampshire, Durham, NH 03824, USA; ^12^Health and Nutritional Sciences Department, South Dakota State University, Brookings, SD 57007, USA

## Abstract

This paper describes the redesign, field-testing, and convergent validity of a practical tool—Physical Activity Campus Environmental Supports (PACES) audit. *Methods.* The audit includes two parts: (1) PACES-Programs, which is comprised of questions regarding populations served, fees, programs (recreation/fitness classes and intramurals), proximity, adequacy of facilities, and marketing, and (2) PACES-Facilities, which is comprised of questions regarding built environment (aesthetics, bike racks, stairs, and universal design), recreation equipment, staff, amenities, and access. Each item criterion is specifically scored using a five-point, semantic-differential scale ranging from limited to extensive environmental support. A few questions utilize select all that apply for a summed score. PACES training, interrater reliability, and data collection are all accessible via an online portal. PACES was tested on 76 college campuses. Convergent validity was examined by comparing the PACES-Programs questions to Healthy Campus Initiatives-Programs questions (HCI-Programs) and comparing the PACES-Facilities questions to questions contained in the Physical Activity Resource Assessment (PARA) Instrument. Statistical analyses included Cronbach's alpha, ANOVA, latent profile analysis, and Spearman correlations. *Results.* The PACES-Programs audit includes 10 items for a potential total of 73 points (*α* = 0.72) and PACES-Facilities audit includes 15 items for a potential total of 77 points (*α* = 0.837). Most (77.8%) of the 153 facilities assessed scored in the most healthful range (20–42), which was mainly due to the extensiveness of the aerobic equipment/amenities and the competence/accessibility of staff. Significant differences in PACES-Total and PACES-Programs scores were associated with campus size and PACES-Facilities across regions. For the paired validation assessments, correlations were significant between PACES-Programs and HCI-Programs ((*n*=41) *r*=0.498, *p* < 0.001) and PACES-Facilities and PARA (*n*=29) for both features (*r*=0.417, *p*=0.024) and amenities (*r*=0.612, *p* < 0.001), indicating moderate convergent validity. *Conclusion.* The PACES audit is a valid, reliable tool for assessing the quality of recreation facilities and programs in a variety of college campus environments.

## 1. Introduction

Obesity prevention guidelines recommend regular physical activity through the lifespan to prevent disease and promote good health [1–3]. The availability, access, quality, and usage of recreation facilities and programs have been identified as factors influencing a population's level of physical activity [4–8]. For school children, the number of outdoor facilities at school was associated with higher physical activity levels [9], and adolescents were more active using public recreation spaces (rather than private) or open field times [10, 11]. On college campuses, recreation facility usage was related to favorable health indices [12]. However, student participation in campus recreation programs declined when membership fees were charged to use the facility, highlighting financial considerations as a barrier to achieving physical activity [13]. Given the basic relationship between the availability of recreation facilities and physical activity levels, the quality and extensiveness of recreation programs and facilities require further study.

The existing tools available to evaluate the quality of recreation facilities/programs are limited. The Worksite Health Promotion Readiness Checklist, a simple yes/no survey, is available to assess the health promotion and protection practices and policies in worksites [14]. The SPOTLIGHT virtual audit tool assesses the presence of indoor/outdoor recreation facilities and public parks via the street view feature of Google Earth/GIS [15]. Another tool based upon Total Quality Management (TQM) evaluates recreation facilities from a variety of user viewpoints, with a focus on safety, condition, and maintenance [16]. This tool can be used to evaluate recreation centers, parks, playgrounds, aquatic facilities/pools, and sports fields, and it was found to be a reliable and effective measure of the physical features (amenities) of a recreation facility [16].

A variety of tools are available to assess students, employees, alumni, and the community satisfaction or perception of recreation services [17–21]. One tool [17] evaluates students' satisfaction levels, perceived service quality, and behavioral intentions for continued use of campus recreation facilities and programs. Using a Likert scale, the topics include facility ambiance, operations quality, staff competency, overall satisfaction, and behavioral intentions. Another tool evaluates students' perceptions of the effectiveness of, and their satisfaction with, campus recreation programs [18]. It assesses personal treatment, budget, academic support, individual performance, and ethics. Using a Likert scale, another tool named “the Scale of Service Quality in Recreation Sports” assesses recreation program client perceptions of quality and satisfaction [19]. Specifically, this tool assesses quality based on the range of programs, operating times, client-employee interactions, interclient interactions, physical changes, valence, social ability, ambient conditions, design, equipment, and satisfaction ratings of programs [19]. A study of students' perceptions indicated that recreation program administration and promotion were important factors because many of the students were unaware of the existence of the available recreation programs [20]. The study also showed that students' perceptions of recreation facilities available on campus differ between men and women and by class standing [20]. Some tools for evaluating recreation facilities are too simple [14] because they primarily assess presence/availability [15] and safety [16] and rely upon client perceptions or satisfaction of facility users [17–21]. Few tools objectively evaluate recreation facilities [22–24].

A Recreational Facility Audit Tool (RecFAT), created and tested in Hong Kong, uses a 111-item checklist to evaluate the availability and accessibility of sport facilities and amenities, policies, environmental safety and aesthetics, and population usage of the facilities [23]. The tool was determined to be reliable and useful for evaluating parks, play grounds, and sports centers. The Physical Activity Resource Assessment (PARA), which has good reliability, was designed to assess publicly available facilities in low-income communities [22]. Trained researchers objectively assess parks, churches, schools, sports facilities, fitness centers, community centers, and trails based on location, cost, features, amenities, qualities, and incivilities (noise, trash, vandalism, etc.). PARA is a general checkoff that evaluates components for presence/quality on a scale of 0 to 3; however, some of the detailed features such as staffing, weight/aerobic equipment, and universal access of recreation center/gym are not assessed.

A tool for objectively evaluating the quality and extensiveness of the recreation facilities is the Physical Activity Campus Environmental Supports (PACES) audit, which was originally developed to assess the environmental supports for recreation programs and facilities related to physical activity on a university campus [24]. In 2009, the PACES audit was conducted at thirteen universities in the United States by trained researchers. PACES audit categories included built environment (bike racks, health promotion signage, and stairwells) and campus recreation programs (availability and quality of equipment, exercise spaces, courts/fields; availability of health education and intramural programs; and recreation facility hours, staff, and amenities). Data were collected with a simple checkoff paper survey tool. To more effectively evaluate and compare the quality and extensiveness/completeness of campus recreation facilities and programs, the purpose of this study was to update/redesign and validate PACES. The updated PACES will be more accessible for monitoring and evaluating campus recreation facilities and programs, with a more user-friendly online data collection format and scored results.

## 2. Methods

### 2.1. Overview

This paper is divided into two parts. Part one includes (1) development of inventory of items for the redesigned PACES audit; (2) expert, cognitive, and pilot testing; (3) survey analysis and revisions; and (4) field-testing. Part two validates PACES by comparing PACES-Facilities to PARA [23] and PACES-Programs to Healthier Campus Initiatives (HCI) [25]. Data were collected between 2015 and 2017 and analyzed in 2018. This study was deemed exempt by Syracuse University IRB because it was an environmental audit not human subject research.

### 2.2. Part 1: Instrument Development

#### 2.2.1. Development of Inventory Items for the Audit

This audit was designed to rate the quality and extensiveness of recreation facilities and physical activity programs. It can be used for municipalities, worksites, schools, and college campuses to evaluate one venue or to understand a more complete picture of the recreation facilities/programs for a specific environment by evaluating a number of venues. With the improved PACES audit training and data entry online system, users are provided with results compared and benchmarked to a wider sample of data.

Worksite/college campus recreation facilities and programs require periodic evaluation to determine their effectiveness for the population that they serve. The evaluation audits the overall campus environment, and the quality and extensiveness of the physical activity supports which contribute to making healthy physical activity decisions. The initiatives/policy support [26] and walkability/bikeability of a campus [27] were evaluated separately with other audits.

To create the survey questions, a four-step process was used. (1) The team reviewed the original PACES to identify the difficulties and limitations for collecting and interpreting the data. (2) The literature [1, 9, 13, 16–18, 24, 28–42] was reviewed to discover the behavioral and environmental correlates for physical activity and decide which topics to include. The following topics emerged for inclusion: facility updates, aesthetics, amenities, and cleanliness; stairwell and bike access; universal access; staff competence; extensiveness and adequacy of health programs, clubs/intramurals, exercise classes, equipment, fields, courts, and trails; and marketing of programs and fees. (3) For each question, semantic differential or Likert scales were created, based on the literature, to indicate low to high support. (4) Originally, one survey was created to include all relevant campus program/recreation facilities topics. As a result of testing the survey, it was divided into separate facilities and programs surveys because campuses could have more than one recreation facility, but only one overall recreation program (Table 1).

The recreation programs audit contains 13 questions including populations served, fees, programs, proximity, and marketing. The recreation facilities audit contains 20 questions including built environment, equipment, staff, amenities, and access. Each item criterion is specifically scored using a five-point, semantic-differential or Likert scale ranging from limited to extensive environmental support/evidence. A few questions utilize select all that apply for a summed score.

#### 2.2.2. Expert, Cognitive, and Pilot Testing

This audit was cognitively tested with seven research assistants. Each student independently attempted to apply/score the audit for two different facilities. Via a group discussion, each question was discussed to determine interpretation, clarity, and appropriateness of semantic or Likert scales. Five public health/recreation program experts reviewed the audits for content validity. The results from the cognitive testing and expert review improved wording of questions and response items. The audit was pilot tested twice. Changes based on pilot testing included dividing it into two surveys for ease of administration and refining the wording of a few questions/responses (Summer 2014 at SU and Fall 2014 data not shown).

#### 2.2.3. Recreation Facilities Venue Definitions

*Main (Primary) Recreation Facility*. This is the only or primary recreation facility for the population served.

*Secondary/Satellite Facility*. This is a smaller recreation facility that houses a portion or smaller version of the total recreation facilities.

*One Component of Facilities*. This includes single components of recreation facilities, such as a pool or a tennis court.

#### 2.2.4. Field Testing: Audit Administration Procedures

This audit was tested on and near college campuses participating in the Get FRUVED research study (*n*=78). Get FRUVED [43] is a social marketing and environmental change intervention to promote health on college campuses. At each college, the venues to be evaluated were determined by a campus team that identified a representative sample of the recreation facilities (main and secondary/satellite) which were most frequented by the campus population. At a minimum, the team assessed the main facility and approximately 25% of the secondary/satellite facilities within a 1.5-mile radius, depending on the campus. In cases where the served population extensively utilized a facility located beyond the 1.5-mile radius, the audit review team could decide to audit it.

Two different assessments were completed on each campus PACES-Facilities and PACES-Programs audit. A PACES-Facilities survey was completed for each recreation facility on/off campus, whether it was for a main recreation facility, secondary/satellite facility, or one component of a facility. For campus programs, one PACES-Programs survey was completed per campus.

*Training and Interrater Reliability*. Research assistants completed video training and practiced and performed interrater reliability (IRR) exercises. Each participating community had its student researchers complete IRR on two recreation facilities. Interclass correlations > 0.80 were required for each team prior to data collection. Starting in 2017, the IRR procedures were converted to an online quiz.

### 2.3. Analysis

Scores were computed for each PACES-Programs and PACES-Facilities survey. Interclass correlations (ICC) were computed to determine interrater reliability. To compare campuses, a PACES-Total score was computed by adding the PACES-Programs and the PACES-Facilities scores for the main facility evaluated. Cronbach's alpha for PACES-Programs was (*α*=0.720, 10 items) after deleting the question “When was the most recent recreation facility built?” Cronbach's alpha for PACES-Facilities was (*α* = 0.837, 15 items). Differences by campus size and region were determined with ANOVA. To distinguish the quality of recreation facilities between campuses, latent profile analysis (LPA) was applied. LPA categorization allows for the assessment of heterogeneity of the sample based on distinctive characteristics of schools' recreation facilities, which were expected to follow non-normal distributions. Two to five profiles were tested iteratively by using the robust maximum likelihood method and Akaike information criteria (AIC), bayesian information criteria (BIC), entropy, and sample size-adjusted BIC (SSABIC). The uniqueness and interpretability of latent profiles were considered to choose the optimal model [44]. Lower AIC, BIC, and SSABIC values indicate better model fit.

## 3. Results

A total of 153 facilities were assessed on and near 76 campuses. Students were effectively trained to implement the audits with ICC ranges of 0.90 to 0.98 for IRR. Sixty percent of the sample was from medium (*n*=21, 29.2%) to large (*n*=24, 33.3%) schools (Table 2). Most audits were completed in the south (*n*=29, 40.3%) with only 15.3% in the west (Table 2). Most facilities were primary (*n*=152, 53.5%), followed by secondary (*n*=86, 30.3%), and finally, 44 one component/stand-alone facilities (15.5%). The scores ranged from 2 to 42 for facilities across all campuses, with a maximum of 77. PACES-Programs scores ranged from zero to 55 across all campuses of a maximum total of 73 points.

Small schools (500–1000 students) scored significantly lower than the largest schools (>20,000 students) on PACES-Total and PACES-Programs, but not on PACES-Facilities. Although there were no differences in PACES-Total by region, the campuses in the west scored significantly lower than all other regions on PACES-Facilities (Table 3).

**Figure 1:**
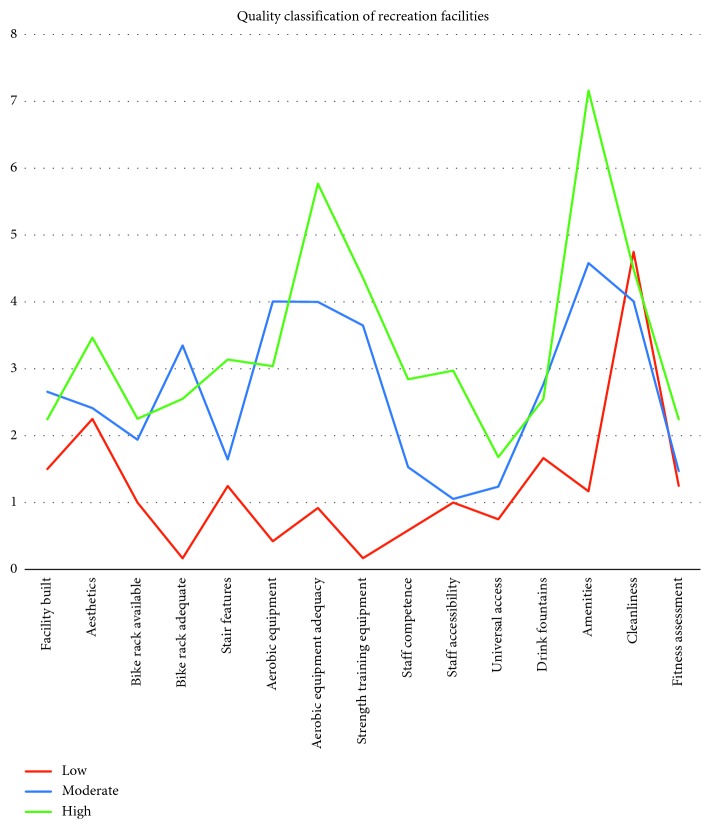
Quality classification of recreation facilities.

The three-class solution was identified as the best model based on AIC (2-class: 9185.638; 3-class: 8954.549; 4-class: 9054.549; and 5-class: 9061.713), BIC (2-class: 9507.555; 3-class: 9428.314; 4-class: 9680.161; and 5-class: 9839.173), and SSABIC (2-class: 9172.049; 3-class: 8934.550; 4-class: 9028.140; and 5-class: 9028.895) values and meaningful interpretation of profiles. Entropy was 0.996 for the three-class solution. The first profile (low quality) consisted of 8.5%, the second profile (moderate quality) consisted of 13.7%, and the third profile (high quality) consisted of 77.8% of the samples (means and ranges available in Table 4).  Figure 1 indicates aesthetics, adequacy of aerobic equipment, staff competence, staff accessibility, and extensiveness of amenities contributed to the facilities classified as high quality. Moderate quality scoring classified facilities scored in the middle for most questions, with moderate peaked scores on bike rack adequacy, aerobic equipment, and amenities. Low quality scoring classified facilities consistently scored lowest on all questions except cleanliness.

The distribution in the quality of the facilities differs by campus size *χ* ^2^ (4, *N*=152 facilities) = 16.994, *p* ≤ 0.01. Approximately 34% of high-quality facilities were in large schools, 34% were in medium size schools, and 25.2% were in the smallest size schools, whereas 61.5% of low-quality facilities were at large schools and 23.1% were in small size schools. Approximately 57.1% of moderate quality facilities were in the smallest schools. No facilities scored in the exceptional quality category.

## 4. Part 2: Convergent Validation Study

### 4.1. PACES-Facilities Validation

#### 4.1.1. Materials

The Physical Activity Resource Assessment (PARA) Instrument [23] was chosen for the validation comparison because it was the most appropriate objective tool to evaluate similar recreation facilities concepts. The survey is a one-page checklist that assesses types of resource, features, amenities, and incivilities. Types of resource (fitness club, park, sport facility, trail, community center, church, school, and combination) are assessed on size, capacity, cost (free, pay at door, pay for certain programs), hours (open and close), and signage (hours, rules: yes/no). For the features and amenity sections of the survey, each item is rated on a 0 to 3 scale: 0 = not present, 1 = poor, 2 = mediocre, and 3 = good. Features include baseball field, basketball court, soccer field, bike rack, exercise station, play equipment, pool >3 feet deep, sandbox, sidewalk, tennis court, trail-running/biking, volleyball court, and wading pool <3 ft. The amenity section includes access points, bathrooms, benches, drinking fountains, fountains, landscaping effort, lighting, picnic tables shaded, picnic tables no shade, shelters, shower/locker room, and trash containers. The incivilities items are rated on a 0–3 scale: 0 = not present, 1 = little/few, 2 = some, and 3 = a lot. Incivilities include auditory annoyance, broken glass, dog refuse, dogs unattended, evidence of alcohol use, evidence of substance abuse, graffiti/tagging, litter, no grass, overgrown grass, sex paraphernalia, and vandalism. Detailed directions were included with the survey.

#### 4.1.2. Protocol

The PACES-Facilities and PARA audits were tested at 29 facilities on and near college campuses (*n*=8). For validation purposes, each auditor/team was responsible for a paired evaluation using both tools (PACES-Facilities and PARA). To reduce potential bias, PARA was collected first for one half of the audits and for the other half, PACES-Facilities was collected first. Auditors entered all surveys into Qualtrics, with one for PARA and one for PACES-Facilities.

*Training and Interrater Reliability*. Research assistants completed training and practiced and performed interrater reliability (IRR) exercises for both tools. Each participating campus had its student researchers' complete IRR on two recreation facilities. Interclass correlations > 0.80 were required for each team prior to data collection.

#### 4.1.3. Analysis

Scoring incivilities were reverse coded: 4 = not present, 3 = little/few, 2 = some, and 1 = A lot. For each section, features, amenity, and incivilities, an average score and a sum score were computed. The reliability for each section was assessed: features (*α*=0.854); amenities (*α* = 0.80); incivilities (*α* = 0.387).

Spearman's correlations were used to compare PACES-Facilities score to the PARA features section and the temporary PACES “amenities items” to the PARA amenities. PACES was not designed to assess incivilities, and the PARA reliability was low, so no comparison was made for this dimension.

### 4.2. PACES-Programs Validation

#### 4.2.1. Materials

To validate PACES-Programs, the results were compared to a survey created from the Partnership for Healthier America's Healthier Campus Initiative (HCI) [25]. A portion of the HCI survey was chosen for this validation because it measures comparable concepts for the college campus regarding extensiveness of health and wellness programming on campus. The HCI survey contained 41 questions, 15 regarding food/nutrition offerings, 19 regarding physical activity programs/facilities, and seven regarding policies. Each question was a Yes/No checkoff to indicate if a campus had the initiative or policy. The 16 specific HCI-programming questions selected for validation included bike share/rental, fitness/intramural opportunities, introduction to physical activity classes, physical activity breaks offered, fitness orientations, sufficient outdoor activities, rental for outdoor equipment, outdoor recreation clinics/trips, marked walking routes, free access to fitness/recreation center, dedicated physical activity space, outdoor running/walking track outdoor fitness system, certified personal trainers, implementation of comprehensive wellness program, and healthy cooking classes.

#### 4.2.2. Procedure

Campuses participating (*n*=78) in Get FRUVED [43] completed PACES-Programs and the HCI survey as part of their full data collection.

#### 4.2.3. Data Analysis

Summing the 16 selected HCI programming questions, the HCI programming subscore was compared to PACES-Programs using Spearman's correlation.

## 5. Validation Results

There were 29 total PACES-Facilities and PARA pairs. Interrater reliability for PARA was ICC = 0.91 to 0.99 and for PACES-Facilities ICC = 0.81 to 1.0. Most of the schools were public institutions (87%) (Table 5). The northeast and the south each represented 30% of the sample, while there were no school facilities evaluated from the west. More than a third of the sample (39%) was from very small and small schools with ≤10,000 students. Correlations were significant between PACES-Facilities (features) and PARA features (*r*=0.417, *p*=0.024) and for PACE-Facilities (amenities) and PARA amenities (*r*=0.612, *p* < 0.001).

Forty-one of the 78 Get FRUVED schools had matched PACES-Programs and HCI data. Most of the schools were public institutions (70.7%) (Table 5). The south represented 39% of the sample, while only 17% were from the northeast. More than a third of the sample (39%) was from very small and small schools with ≤10,000 students. There was significant correlation between total PACES-Programs and the HCI programming subscore (*r*=0.498, *p* < 0.001).

## 6. Discussion

For this study, the team redesigned, tested, and validated an updated version of PACES, a reliable tool to assess the quality of recreation facilities and programs. PACES distinguishes differences between facility types and programs, across campuses and regions. Most facilities evaluated with PACES-Facilities categorized into the highest quality recreation facilities category, primarily due to the extensiveness of their aerobic equipment and amenities and the competence and accessibility of the staff. The range of PACES-Programs scores indicated that most campuses provided a moderate level of options and supports for their overall campus programs.

Only a few researchers have attempted to assess the quality of recreation facilities/programs, with most relying on client or user perception [17–19, 45]. The original PACES [24], PARA [23], and RecFAT [22] more objectively assessed quality by focusing on condition and maintenance. The comparisons between PACES and previous research are limited because the latter typically evaluates only portions of the physical activity environment, such as presence/availability of recreation facilities [15, 46] or user satisfaction [20, 21]. Some existing audits are focused on safety [16], universal access [39], park quality [47, 48], or rural environments [49]. In a systematic review of worksite tools [50] related to supports for physical activity (*n*=15), only 20% of the tools were objective audits while over 50% were based upon employee self-report. Of the tools reviewed, 75% of the studies included access to physical activity equipment/facilities, amenities, and an assessment of educational opportunities; 66% of studies included bike rack availability/stairwell features; and less than 50% included evaluation of fitness assessment opportunities [50]. While 50% had completed internal and/or interrater reliability, only 33% reported some level of validation.

The PARA and RecFAT tools are useful in a diversity of recreation environments and are less specific regarding the details of campus recreation facilities. For this study, PARA was helpful as a validation comparison for PACES-Facilities for features and amenities [23]. For the subsample assessed by both PARA and PACES-Facilities, there were significant correlations for both features and amenities (PACES-Facilities does not assess incivilities, so no comparison was made.) Using PARA, Adamus et al. [51] found some similar results to these found on college campuses: fitness clubs had the highest scores for amenities and combination resources had the highest scores for features.

Others have found low to moderate reliability between population perception and objective (Google Earth) assessment of recreation facilities [52]. PACES can be a valuable and objective tool for evaluating and comparing the quality of recreation programs and facilities in varied environments. This is important because recreation facility quality has been found to relate to a variety of outcomes [6, 7, 9, 10, 21, 53–59]. The accessibility of recreation facilities are related to the level of physical activity in various populations [6, 7, 9, 10, 21, 53, 54, 60], and a community's natural amenities and recreation facilities per capita are negatively related to the populations' rate of obesity [58]. On college campuses, recreation facility usage has been related to higher academic outcomes (GPA) [12, 59], higher student retention [56, 59], reduced stress [57], increased exercise frequency [42], and improved health indices [5]. The quality of recreation services (specifically staff competency, operations quality, and facility ambiance) has been shown to influence levels of satisfaction with recreation facilities and programs [17].

The PACES training and practice require approximately 2-3 hours, and an audit can be completed in 25–30 mins per facility or survey. The PACES audit is part of the Healthy Campus Environmental Audit (HCEA), a series audit tools to evaluate restaurants [61], convenience stores [62], vending [63], walkability/bikeability [27], and policies [26]. The PACES audit is user-friendly and available on the internet, with training and data entry links (contact the primary author for information). The primary institution analyses the data and provides feedback to the user including comparison and benchmark information. PACES-Facilities and PACES-Programs audits have been validated for college campus-type work environments but might also be useful in a variety of settings including communities, worksites, colleges, and schools.

A limitation of this study is that only college-educated populations on and near college campuses have used and tested the audit, and it therefore needs to be validated for use by other data collectors to determine the utility of PACES in communities beyond college campuses. Although many recreation facilities audits are perception-based [17–21, 45], and some contradict objective findings [52], the PACES audit should be further tested by comparing audit results with recreation facilities/program clients' perceptions. Future research should also evaluate the relevance and weighting of PACES items and the effectiveness of facilities and program supports in encouraging physical activity.

## Figures and Tables

**Table 1:** PACES audit questions.

Categories	Questions	Scoring
	PACES-Programs	
Population	Subpopulations with access to recreation facilities and programs: (i) Students, employees, employee's families, community, and alumni	Select all that apply

Fees	Additional fees for recreation services and programs [13]: (i) Fees for fitness classes, fitness center, intramurals, and sports clubs (ii) Note: recreation fees integrated in the student tuition do not classify as an additional fee (iii) Employees and students are assessed separately	Select all that apply

Facilities (*n*=4)	When was the most recent recreation facility built? [42] (i) Answers: >15 years, 11–15 years, 6–10 years, 1–5 years, and new facility	Question specific
	How far is the closest walking/biking trail from the geographic center of campus? [34, 41] (use Google Maps to determine distance) (i) Length of trail must be at least ½ mile in length; Can be circuit or non-continuous; Does not have to be scenic or attractive; Trail can be through campus or city as long as it is a marked trail (ii) Answers: no trail, > 1 mile from center, 2/3–1 mile, 1/3–2/3 mile, and < .3 mile	Question specific
	Indoor/outdoor facilities available for all student/faculty not just athletes [9, 24] (i) All-purpose (lacrosse, soccer, etc.), baseball/softball, basketball, football, tennis, track, skating rink, volleyball, pool, and other	Select all that apply
	Adequacy of indoor/outdoor facilities based on availability, condition, size, and sufficiency for the campus population [16]	SD to SA^1^

Programs (*n*=5)	How many health/wellness activities and events are offered for Tuesday and Wednesday (a representative sample)? [24, 32, 40] (i) Evaluator should count the amount of health-related offerings (events, lectures, guest speakers, workshops, outings, free fitness classes, and group sports) on entire campus for each day	1-2 up to > 10^2^
	How many different varieties of fitness classes are available? [24, 32] (i) Do NOT combine classes offered in the spring and fall. Assess classes available for one-semester, preferably in the current semester. If your campus does not operate by semesters, evaluate classes available over the prior 4 months	1-5 varieties up to > 25^2^
	Intramurals and club sports [18, 24] (i) Select all choices that apply: Variety of subgroups within sports (i.e. men, women, Greek, recreational, competitive, faculty), variety of sports offered, ability for groups to create teams, ability to waitlist if all team slots are filled, ability to create/add teams to meet demand during the season Adequacy of intramural and club sports [16] (ii) Adequacy should be based upon the amount of choices above selected	Select all that apply^2^ & SD to SA^1,2^
	How are recreation programs reserved? [33] (i) Programs can include but not limited to fitness classes, personal training, club sports, intramurals, excursions, etc. (ii) Answers: first come first serve, paper-based, computerized, and call-based reservation	Select all that apply

Proximity	How many residence halls are within 2/3 miles of the geographic campus center? [38] (use Google Maps to determine the distance) (i) Answers: none, 1, 2, 3, and >3 residence halls	Question specific^2^

Marketing	How frequently is social media updated to promote recreation facilities and programs? [30] (i) Answers: no social media exists, sporadic, 1 update daily, 2 updates daily, >2 updates daily	Question specific
	PACES-Facilities	
Built environment (*n*=6)	When was the recreation facility built? [42] (i) Answers: >15 years, 11–15 years, 6–10 years, 1–5 years, and new facility	Question specific
	Recreation facility aesthetics and building context [31] (i) Answers include windows providing an outdoor view are present in the recreation area, building is free-standing, separated from other buildings in the proximity, closest building should be at least 200 feet away, and attractive view from inside facility	Select all that apply
	Bike racks: availability [24, 37] (i) Note: all possible entrances to facility must be evaluated (ii) Answers: no racks, rack by 1, 2, 3, and ≥4 entrances.	Question specific
	Bike racks: adequacy [16] (i) Answers: 0–20%, 21–40%, 41–60%, 51–80%, and 81–100% spots available	Question specific^2^
	Stair features [24, 29] (i) Answers: centrally located, safety features, aesthetically pleasing, signage, and accessible Definitions: (a) *Centrally located*. Stairs being visible from the front entrance of building (b) *Accessible*. Unlocked stairs and stair width sufficient for 2 people (c) *Aesthetically pleasing*. Creative lighting, decorative, carpeted, bright colored walls, artwork, motivational signs, and music (d) *Safety*. Well lit, rubber treading on steps (slip resistant), and hand rail fully extended length of stairs (e) *Signage*. Signage to steps, absence of emergency exit ONLY label/sign, and numbered floors in stairwell (f) Stairs should be assessed for the primary recreational facility and secondary recreational facility and any components of a recreational facility	Select all that apply^2^
	Universal design features [39] Answers: (1) Exercise equipment is available that does not require transfer from wheelchair to machine (2) Pool lift controls accessible from the deck level for individuals that use a wheelchair. The pool has a ledge to hold on to when entering the water (3) Is a customer's personal assistant allowed to enter the facility without incurring additional charges?	Select all that apply

Equipment (*n*=4)	Aerobic equipment: available equipment types [24, 28] (i) Answers: treadmill, bike, air rower (rowing machine), stair stepper, cycle ergometer, and other (list)	Select all that apply^2^
	Aerobic equipment: accessibility [24] (i) Answers: 0–19%, 20–39%, 40–59%, 60–79%, and 80–100% spots available	Question specific^2^
	Strength training equipment: available equipment types [24, 35] (i) Answers: Resistance machines, free weights, barbells, at least 100 square ft. of open space, and raised platforms	Select all that apply^2^
	Equipment scheduling [33] (i) Reservation for recreation equipment should be found within recreational facility or online (ii) Recreation equipment can include but not limited to cardiovascular machines, multipurpose rooms, resistance equipment, etc.	
Staff (*n*=2)	Staff competency [17, 24] (i) Note: inform the staff member that you are conducting an audit; ask him/her to show you around and to tell you where all of the equipment is located (i.e., aerobic and strength training equipment); assess if the staff member provided assistance in a professional manner, made eye contact, and was able to provide guidance in regards to the function and use of the equipment (ii) Answers: staff not able to assist, staff willing to assist but could not provide accurate guidance, and staff was willing to assist and provided accurate guidance Staff accessibility [10, 16] (i) Answers: no staff present, staff present but unavailable or busy with other customers, and staff present and available	Question specific

Amenities (*n*=4)	Drinking fountains [16, 24] (i) Answers: no drinking fountains, drinking fountains, and refillable bottle stations available	Question specific
	Amenities [16] (i) Answers: locker rooms, lockers outside locker room, showers, hand towels, televisions, reading material, hand sanitizer, music, disinfectant spray, and other (list)	Select all that apply
	Cleanliness [17, 24] (i) The following areas (if available) are clean (no trash present): restrooms, weight room, locker room, activity courts (all purpose), indoor track, racquetball courts, entrance/hallways, Pool(s), and outside recreation facility	SD to SA^1^
	Is an initial fitness assessment offered? [1, 24] (i) Answers: no fitness assessment, additional charge for fitness assessment, fitness assessment is mandatory, fitness assessment at no charge, fitness assessment provided with workout plan/recommendation	Select all that apply

Access	Number of hours facility is open [24, 36] (i) Hours of operation are assessed for Tuesday, Saturday, and Sunday	0 to 24 hours

Responses required: ^1^Likert scale defined: strongly disagree, disagree, neutral, agree, and strongly agree; ^2^not applicable and does not apply to our environment.

**Table 2:** Sample distribution.

*Campus population*	*N*	*Percentage*	
Very small ≤5000	18	25	
Small 5001–10,000	9	12.5	
Medium 10,001–20,000	21	29.2	
Large ≥20,000	24	33.3	

*Campus location*	*N*	*Percentage*	
Northeast	14	17.4	
Midwest	18	25	
South	29	40.3	
West	11	15.3	

*Survey scores*	*N*	*Mean ± SD*	*Range*
*On campus*			
PACES-Programs	76	38.22 ± 13.86	0–55
PACES-Facilities: primary	128	28.65 ± 8.43	5–42
PACES-Facilities: secondary	71	21.23 ± 8.93	2–38
PACES-Facilities: single	44	14.34 ± 6.65	2–31

*Off campus*			
PACES-Facilities: primary	25	26.32 ± 6.38	10–39
PACES-Facilities: secondary	15	23.26 ± 6.87	2–31

**Table 3:** Differences in PACES-Total, PACES-Programs, and PACES-Facilities by campus size and region.

Campus size^2^	Total^1^		Programs		Facilities	
	*N*	Mean ± SD	*N*	Mean ± SD	*N*	Mean ± SD
Very small	18	63.19 ± 19.05^ab3^	18	37.03 ± 7.88^ab4^	41	27.90 ± 5.31^5^
Small	9	59.43 ± 13.16^a^	9	35.69 ± 5.55^a^	15	25.33 ± 10.60
Medium	21	77.48 ± 19.49^ab^	21	42.18 ± 7.81^bc^	46	30.00 ± 6.93
Large	24	79.21 ± 24.22^b^	24	46.82 ± 5.18^c^	50	27.92 ± 30.07

*Region*						
Northeast	14	75.44 ± 23.49	14	40.44 ± 7.85	46	29.09 ± 5.03^c6^
Midwest	18	73.32 ± 22.62	18	41.64 ± 7.78	34	30.20 ± 7.04^c^
South	29	71.52 ± 21.24	29	42.69 ± 7.85	51	28.84 ± 7.60^c^
West	11	68.23 ± 21.15	11	40.80 ± 9.34	21	22.09 ± 13.19^d^
Total	72	72.23 ± 21.67	66	41.69 ± 7.93	152	28.66 ± 8.43

^1^PACES-Total = PACES-Programs + PACES-Facilities (for the main facility audited).^2^Campus size based upon student population: very small ≤5000; small 5001–10,000; medium 10,001–20,000; large ≥20,001. ^3^ *F* = 3.715, df = 3, *p*=0.015; different subscripts are significantly different. ^4^ *F* = 8.779, df = 3, *p*=0.0001; different subscripts are significantly different. ^5^ *F* = 1.397, df = 3, *p* > 0.05. ^6^ *F* = 5.264, df = 3, *p*=0.002; different subscripts are significantly different.

**Table 4:** Quality score classification for all main facilities (on and off campus).

Classification	*N*	Mean ± SD	Range
Low	13	7.69^a^ ± 2.35	5–11
Medium	21	22.47^b^ ± 4.03	16–30
High	119	31.54^c^ ± 4.36	20–42
Total	153	28.27 ± 8.16	5–42

*F* = 213.61, *P* ≤ 0.0001; different subscripts are significantly different.

**Table 5:** Characteristics of schools participating in the validation study.

School characteristics	PACES-Facilities		PACES-Programs	
	Frequency		Frequency	
	*N*	%	*N*	%
Private	1	13	12	29.3
Public	7	87	29	70.7

*Geography*				
Northeast	3	30	7	17
Midwest	2	25	9	22
South	3	30	16	39
West	0	0	9	22

*Campus size* ^a^				
Very small	0	0	10	24.4
Small	0	0	6	14.6
Moderate	4	50	12	29.3
Large	4	50	13	31.7

^a^Campus size based upon student population: very small ≤5000; small 5001–10,000; medium 10,001–20,000; large ≥20,001.

## Data Availability

Data are available upon request to the corresponding author.
